# Extra cup of tea intake associated with increased risk of Alzheimer’s disease: Genetic insights from Mendelian randomization

**DOI:** 10.3389/fnut.2023.1052281

**Published:** 2023-01-25

**Authors:** Yuxuan Sun, Zixin Liang, Xiaoxuan Xia, Maggie Haitian Wang, Chengming Zhu, Yihang Pan, Rui Sun

**Affiliations:** ^1^Scientific Research Center, The Seventh Affiliated Hospital, Sun Yat-sen University, Shenzhen, China; ^2^Clinical Big Data Research Center, The Seventh Affiliated Hospital, Sun Yat-sen University, Shenzhen, China; ^3^Department of Statistics, The Chinese University of Hong Kong, Hong Kong, Hong Kong SAR, China; ^4^The Jockey Club School of Public Health and Primary Care, Faculty of Medicine, The Chinese University of Hong Kong, Hong Kong, Hong Kong SAR, China

**Keywords:** tea intake, Alzheimer’s disease, brain volume, SNP, Mendelian randomization

## Abstract

**Background:**

Observational studies report inconclusive effects of tea consumption on the risk of Alzheimer’s disease (AD), and the mechanisms are unclear. This study aims to investigate the effects of genetically predicted tea intake (cups of tea consumed per day) on AD, brain volume, and cerebral small vessel disease (CSVD) using the two-sample Mendelian randomization (MR) method.

**Methods:**

Summary statistics of tea intake were obtained from UK Biobank (*N* = 447,485), and AD was from the International Genomics of Alzheimer’s Project (*N* = 54,162). Genetic instruments were retrieved from UK Biobank using brain imaging-derived phenotypes for brain volume outcomes (*N* > 33,224) and genome-wide association studies for CSVD (*N*: 17,663–48,454).

**Results:**

In the primary MR analysis, tea intake significantly increased the risk of AD using two different methods (OR_IVW_ = 1.48, 95% CI: [1.14, 1.93]; OR_WM_ = 2.00, 95% CI: [1.26, 3.18]) and reached a weak significant level using MR-Egger regression (*p* < 0.1). The result passed all the sensitivity analyses, including heterogeneity, pleiotropy, and outlier tests. In the secondary MR analysis, per extra cup of tea significantly decreased gray matter (β_WM_ = −1.63, 95% CI: [−2.41, −0.85]) and right hippocampus volume (β_WM_ = −1.78, 95% CI: [−2.76, −0.79]). We found a nonlinear association between tea intake and AD in association analysis, which suggested that over-drinking with more than 13 cups per day might be a risk factor for AD. Association analysis results were consistent with MR results.

**Conclusion:**

This study revealed a potential causal association between per extra cup of tea and an increased risk of AD. Genetically predicted tea intake was associated with a decreased brain volume of gray matter and the right hippocampus, which indicates that over-drinking tea might lead to a decline in language and memory functions. Our results shed light on a novel possible mechanism of tea intake to increase the risk of AD by reducing brain volume.

## Graphical Abstract

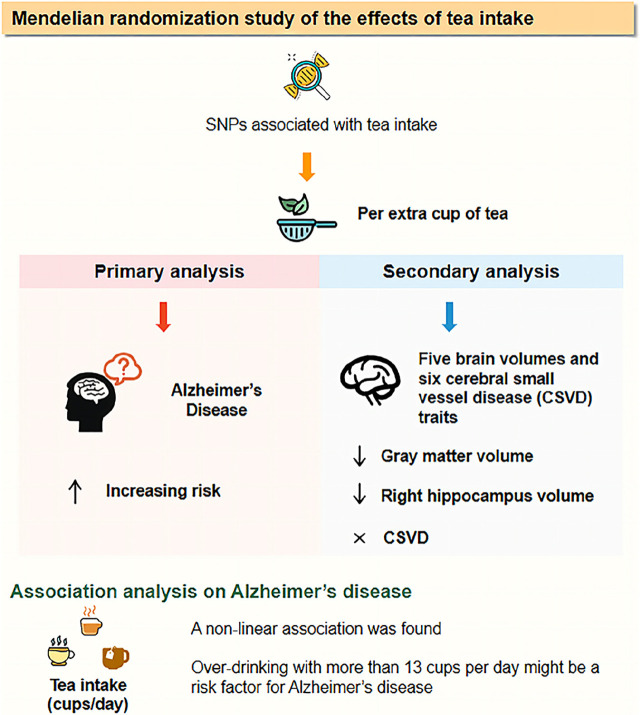

## Introduction

Alzheimer’s disease (AD), the most cause of dementia, is a neurodegenerative disorder associated with cognitive declines and progressive memory loss that subsequently causes language deficiencies accompanied by behavior disorders (1, 2). Approximately 55 million people live with AD or related forms of dementia worldwide (3), and the number of AD cases keeps increasing due to the aging population (4). By 2050, 1 in 85 persons will be living with AD worldwide, escalating the risk of disability, the burden of illness, and health care costs (5). However, AD treatment options are very limited and could only provide intermediate or temporary maintenance of cognitive functions without altering disease progression (6). Foods, physical activities, and other modifiable risk factors are suggested to be potent interventions or caring strategies for AD.

Tea, one of the most popular non-alcoholic beverages, has attracted lots of interest for its possible protective effects on body health (7); however, its effect on AD is unclear. The catechin polyphenols in green tea have neuroprotective effects such as inhibition of amyloid-beta aggregation and anti-apoptosis, which suggests that green tea may have significant protective effects on AD (8). A cross-sectional survey of 2015 subjects aged 65 or older in Eastern China found that tea consumption was associated with a low prevalence of AD and severe cognitive impairments (9). However, the association between tea intake and AD remained inconclusive. Studies on caffeine, another major component in tea, indicated that tea might not have benefits and even have risk effects on AD. For example, a meta-analysis of 20 studies comprising 31,479 subjects found that caffeine intake from coffee or tea was not associated with the risk of cognitive disorders (10). A study on 434,900 UK Biobank participants showed that recent caffeine use reduced performance on prospective memory, pair matching, and fluid intelligence, which suggested potential impairments in memory and reasoning (11).

The results from observational studies are often limited by measurement errors, confounders, and reverse causality. Exposures were identified as risk factors and proposed to be valuable interventions but later proven non-causal. Randomized trials may need decades to produce robust results since some exposures, such as diet, may take years to affect diseases (12). Meanwhile, because of the nature of some diseases like cardiovascular diseases and exposures such as smoking, sometimes, it may be unethical or not feasible to perform randomized trials (13). According to Mendel’s law of independent assortment, Mendelian randomization uses genetic variants as a proxy for exposure, and the inheritance between these genetic variants and other traits is independent. Thus, it overcomes limitations in observational studies and produces relatively robust causal inferences indicating the long-term effects of the exposure (12, 14).

This study conducted a Mendelian randomization analysis to assess the causal relationship between tea intake and AD. Cerebral small vessel diseases (CSVD) and brain volume are closely related to both the presence and the severity of the clinical symptoms of AD (15). In order to better understand the effects of tea intake on AD, we further explored the effects of tea intake on six CSVD and five brain volume outcomes using the two-sample Mendelian randomization method. To further ascertain whether the relationship between tea intake and AD is linear or not, we performed two association analyses of tea intake and AD in UKB cohorts. Findings from this study will reveal the possible causal relationships of tea intake with AD and related traits. This might give clues on practical prevention strategies for AD patients.

## Materials and methods

### Two-sample Mendelian randomization

#### Genetic instruments related to tea intake/consumption

**FIGURE 1:**
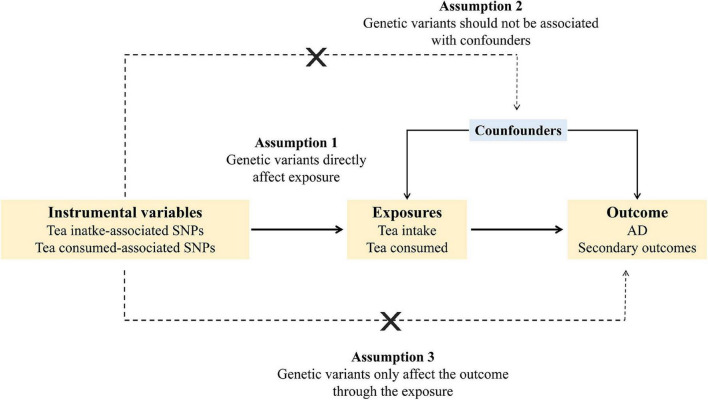
Schematic diagram of the Mendelian randomization assumptions. AD, Alzheimer’s disease.

A valid instrumental variable needs to satisfy three assumptions (Figure 1). First, the genetic variants should be associated with the exposure. Second, the genetic variants should not be associated with confounders. Third, the genetic variants should only affect the outcome through the exposure.

Tea intake measured by cups of tea consumed per day (UKB Data-field ID: 1488), and tea consumed described as whether consumed tea yesterday (UKB Data-field ID: 100309) were two exposures. Summary-level data on tea intake (*N* = 447,485) and tea consumed (*N*_cases_/*N*_controls_ = 51,690/13,259) was obtained from MRC Integrative Epidemiology Unit (IEU) open genome-wide association study (GWAS) project using GWAS ID ukb-b-6066 and ukb-b-17988^1^ (16). Genetic instruments were selected using a moderate genome-wide significance (*p* < 5 × 10^–7^). After that, SNPs were clumped with linkage disequilibrium within 1 Mb genomic distance (*R*-squares > 0.001) using the 1,000 Genome European reference panel and SNPs were excluded if not presented in the outcome GWAS (17). A total of 87 SNPs were selected as genetic instruments for tea intake and three SNPs for tea consumed. To avoid potential confounding, we checked each instrument Single-nucleotide polymorphism (SNP) in the PhenoScanner GWAS database^2^ (18, 19). Four SNPs (rs11164870, rs34619, rs9302428, and rs6129076) were associated with illiteracy, which described as “college or university degree and qualification: none of the degrees” in the original GWAS (20), and were recognized as potential confounders to AD. These four SNPs were excluded from the analysis. Finally, a total 84 SNPs were eligible genetic instruments for tea intake and two SNPs for tea consumed. Detailed SNP information used in primary analysis were in Supplementary Table 1.

#### Genetic variants related to the primary outcome: AD

Alzheimer’s disease was the primary outcome of our analysis. We used a two-sample MR method to assess the causal association between two phenotypes of tea consumption and AD separately. We obtained summary statistics of AD from the first stage of the International Genomics of Alzheimer’s Project (IGAP) (21). The first stage of IGAP meta-analyzed four GWAS datasets: (1) Alzheimer’s Disease Genetics Consortium (ADGC), (2) the Cohorts for Heart and Aging Research in Genomic Epidemiology consortium (CHARGE), (3) the European Alzheimer’s disease Initiative (EADI), and (4) the Genetic and Environmental Risk in AD consortium (GERAD). A total of 17,008 cases and 37,154 controls of European ancestry were included in the study. All studies included in IGAP were approved by a relevant Institutional Review Board.

Replication analysis was performed using data of a meta-analysis for late-onset AD (LOAD) on 13 cohorts excluded 23andMe conducted by Psychiatric Genomics Consortium based on European and US populations (22). This meta-analysis identifies risk loci of LOAD with 43,725 clinical cases and 717,979 controls, as well as 46,613 proxy cases and 318,246 proxy controls from UK Biobank. Informed consent was obtained from all participants.

#### Genetic variants related to secondary outcomes: MRI markers of brain volumes and cerebral small vessels disease

For secondary outcomes, we performed the MR analysis only using exposure that was significantly associated with our primary outcome.

##### Brain volume traits

We included total brain volume (gray matter and white matter), gray matter volume, white matter volume, left hippocampus volume, and right hippocampus volume in the study. Summary statistics of brain volume traits were obtained from a GWAS of brain imaging-derived phenotypes in up to 33,224 individuals from the UK Biobank (23). The brain volume was measured by magnetic resonance imaging, and the phenotypes of brain volume were quantile-normalized. Confounders such as age, sex, head size, and head motion were adjusted.

##### Cerebral small vessels disease/trait

In the secondary analyses, we included the mean diffusivity (MD) and fractiona anisotropy (FA) (*N* > 17,663), white matter hyperintensity (WMH) volumes, and three types of brain microbleeds (BMBs), including any BMBs (*N* = 3,556), strictly lobar (*N* = 2,179), and mixed types (*N* = 1,293). Mean diffusivity and fractional anisotropy were obtained from a genetic association study of small vessel disease using MRI markers (24). WMH volumes data were obtained from a large multi-ancestry GWAS meta-analysis combining samples from the Cohorts for Heart and Aging Research in Genomic Epidemiology (CHARGE) Consortium and the UK Biobank (25). We accessed the WMH data *via* the GWAS catalog^3^ using accession number GCST011947. Only European ancestry data were used in our analysis (*N* = 48,454). Data on BMBs were obtained from a GWAS meta-analysis of 11 population-based cohort studies and three case-control or case-only stroke cohorts (26). All GWAS for secondary outcomes were adjusted for age, sex, and population structure or batch effects.

#### Statistical analysis

Three MR methods were applied to estimate causal effects: inversed-variance weighted (IVW) regression, weighted median (WM), and MR-Egger regression. IVW regression was used to derive causal estimates where at least two exposure SNPs were available for analysis. The WM method would have a lower bias than the IVW method, which makes the causal inference more robust when the pleiotropy assumption is not satisfied (27). The MR-Egger test was used to assess horizontal pleiotropy since the intercept of MR-Egger represents the average horizontal pleiotropic effect across the exposure genetic instruments, where the slope of the regression was the causal estimate (28).

Sensitivity analyses were performed using the MR-Egger intercept test, MR pleiotropy residual sum and outlier (MR-PRESSO), and Cochran’s Q test. MR-Egger intercept test and MR-PRESSO global test were used to assess horizontal pleiotropy, and the MR-PRESSO method could also check and correct for outliers. Cochran’s Q test was applied to detect heterogeneity. The Bonferroni correction method was used for multiple testing in the secondary MR analysis, and *p*-values less than 0.005 (0.05/11) were considered significant. MR analyses were performed in R 4.1.0 (29) using TwoSampleMR (30), MendelianRandomization (31), and MRPRESSO (32) packages. Meanwhile, we used an online calculator for the power analysis (33).

Association analysis between tea intake and Alzheimer’s disease was evaluated using a generalized linear regression model. Participants were divided into eight categories according to tea intake amount, including non-drinkers as well as drinkers consuming less than 1 cup/day, 1–2 cups/day, 3–4 cups/day, 5–6 cups/day, 7–8 cups/day, 9–10 cups/day, 11–12 cups/day, and ≥13 cups/day. Binary AD data and polygenic risk scores (PRS) of AD from UKB were used in the regression model. Binary AD outcome was derived using ICD-10 codes (UKB field-ID: 41202) and self-reported non-cancer illness (UKB field ID: 20002). To better fit the model, we only included participants >65 years old from European ancestry. We used the PRSice2 software (34) to generate the PRS of AD. Quality control and clumping were performed on the genotype data. Additive genetic model were used as the regression model. To adjust for population stratification and other confounders, we included the top 10 principle components, age and sex as covariates in the regression model when generating the PRS. Logistic regression model for AD and linear regression model for PRS of AD were fitted correspondingly to examine the association between tea intake and AD with non-drinkers and drinkers consuming less than 1 cup/day as a reference group adjusted by age at baseline, sex, education, body mass index (BMI), smoking status, alcohol drinking status, total vegetable intake, total fruit intake, total fish intake, sleep duration, and Townsend deprivation index (TDI). Detailed description of derivation of binary AD outcome, the PRS calculation, and association models can be found in the Supplementary Note 1.

## Results

**FIGURE 2:**
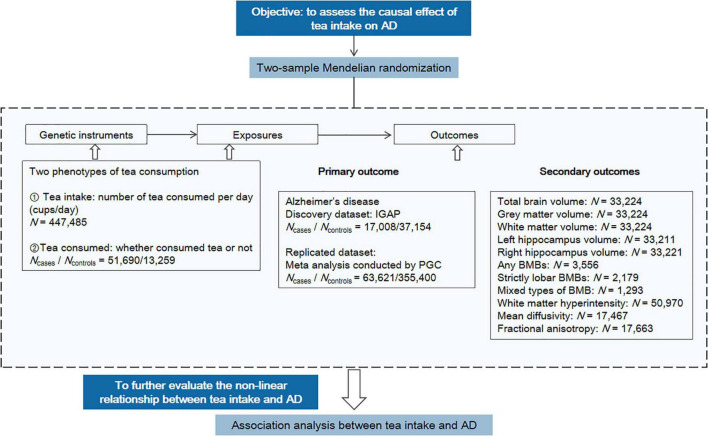
Overview of the study design. The workflow summarized the information of datasets, exposures, and outcomes we used in the primary and secondary MR analyses. AD, Alzheimer’s disease; PGC, Psychiatric Genomics Consortium; BMB, brain microbleed.

The study design is shown in Figure 2, including the primary MR analysis, secondary MR analysis, and association analysis for evaluating potential nonlinear associations.

### Two-sample MR analysis of tea intake and AD or related outcomes

#### The primary MR analyses unveiled novel causal effects between tea intake and AD

**FIGURE 3:**
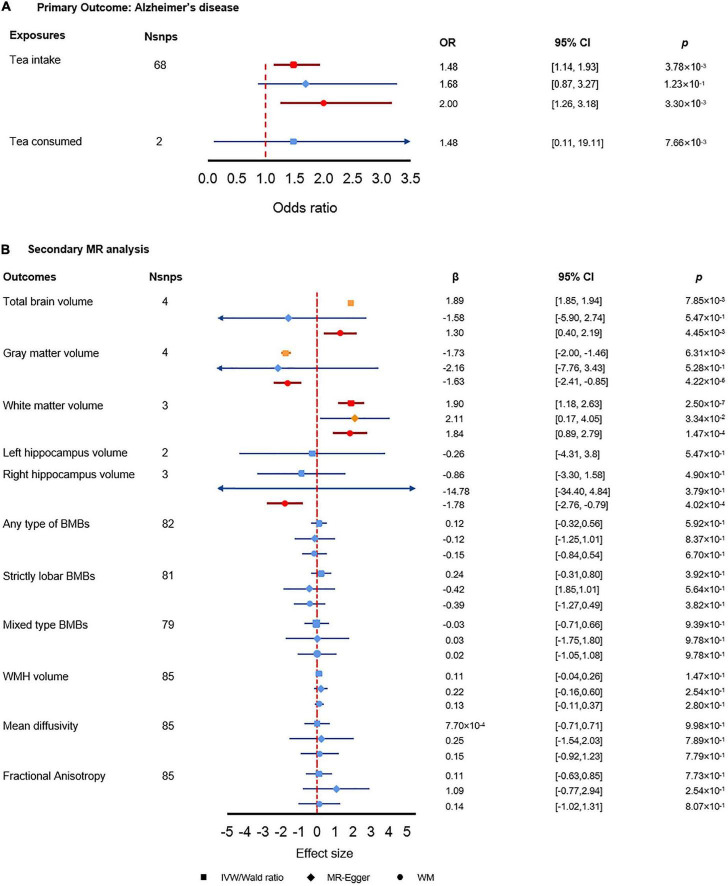
Results of the primary and secondary Mendelian randomization (MR) analysis. **(A)** Primary MR analysis results. **(B)** Secondary MR analysis results. The red color denotes the significant result, the orange color denotes results with *p*-value less than 0.05 but did not pass the multiple test correction criteria, and the blue color denotes insignificant results.

As shown in Figure 3A, genetically predicted tea intake was significantly associated with AD using either IVW estimate or WM estimate (OR_IVW_ = 1.48, 95% CI: [1.14, 1.93], *p* = 3.78 × 10^–3^; OR_WM_ = 2.00, 95% CI: [1.26, 3.18], *p* = 3.30 × 10^–3^). The MR-Egger regression estimate showed close to significance and converging results as IVW estimate and WM estimate (OR_MR–Egger_ = 1.68, 95% CI: [0.87, 3.27], *p* = 1.23 × 10^–1^). For each extra cup of tea, the odds of AD would be greatly increased by more than 1.48 folds. Along with the insignificant but increasing risk effect of tea consumption, the results showed that per extra cup of tea intake might increase the risk of AD. Sensitivity analysis did not find any pleiotropy effect or heterogeneity, which further indicated the reliability of our results. The analysis had 97% power for detecting the causal association (Supplementary Table 2).

In the replication data sets, we did not find a significant association between tea intake and AD. However, the estimated effect direction of all the three MR methods consistently indicated that tea intake was associated with an increased risk of AD, which matched the direction of effect of tea intake we found in the primary analysis (Supplementary Table 3). MR-Egger intercept test and Cochran Q test did not find evidence of horizontal pleiotropy and heterogeneity. The MRPRESSO global test indicated there could be pleiotropy, which may lead to the potential bias of the estimates (Supplementary Table 3).

#### Secondary MR analysis further reveals causality between tea intake and brain volume traits

We found significant associations between tea intake and brain volumes (Figure 3B). First, tea intake was significantly associated with increased total brain volume (β_WM_ = 1.30, 95% CI: [0.40, 2.19], *p* = 4.45 × 10^–3^). Secondly, tea intake was associated with decreased gray matter volume (β_WM_ = 0.20, 95% CI: [0.09, 0.43], *p* = 4.34 × 10^–5^) while tea intake was significantly associated with increased white matter volume (β_IVW_ = 1.90, 95% CI: [1.18, 2.63], *p* = 2.50 × 10^–7;^ β_WM_ = 1.84, 95% CI: [0.89, 2.79], *p* = 1.47 × 10^–4^). The opposite effect directions of tea intake on gray and white matter volumes may elucidate how tea intake affects AD *via* brain volumes. Meanwhile, we also found that tea intake was strongly associated with decreased right hippocampus volume, which could explain the interaction between tea intake and AD (β_WM_ = −1.78, 95% CI: [−2.76, −079], *p* = 4.02 × 10^–4^). We did not find any significant association between tea intake and other secondary outcomes.

The MR-Egger intercept test did not find pleiotropy in any of the associations (Supplementary Table 4). However, MRPRESSO global test found pleiotropy in total brain volume (*p* = 1.91 × 10^–2^), gray matter volume (*p* = 1.91 × 10^–2^), and fractional anisotropy (*p* = 1.91 × 10^–2^), which suggested potential bias to the MR-Egger estimates. The Cochran’s Q test found heterogeneity in the association with total brain volume (*p* = 8.87 × 10^–5^), gray matter volume (*p* = 4.35 × 10^–5^), right hippocampus volume (*p* = 4.71 × 10^–3^), and fractional anisotropy (*p* = 1.98 × 10^–2^). The power analysis showed that the traits with significant results in secondary analysis had enough power of detecting the causal estimates (Supplementary Table 2).

### Association analysis between tea intake and Alzheimer’s disease in UK Biobank cohort

#### Generalized Linear regression found a nonlinear association between AD and tea intake

**FIGURE 4:**
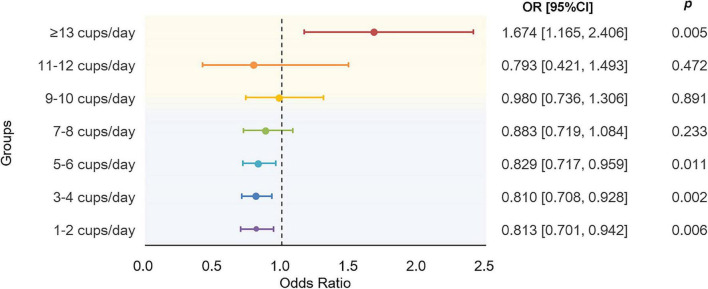
Results of association analysis using a generalized linear regression model. The model were adjusted by age at baseline, sex, education level, BMI, TDI, sleep_duration, total vegetable intake, total fruit intake, total fish intake, smoking status, and alcohol drinking status. BMI, body mass index; TDI, Townsend deprivation index.

The results using binary AD as the response showed a nonlinear association between tea intake and AD (Figure 4). Compared to non-drinkers, people who drink a moderate amount of tea per day had decreased risk of AD, especially in people who drink 5–6 cups of tea per day (OR = 0.83, 95% CI: [0.72, 0.96], *p* = 1.14 × 10^–2^). As the amount of tea intake increases, the protective effects are gradually dismissed and eventually become harmful. As shown, instead of protective effects, people who drink equal to or more than 13 cups of tea per day would have dramatically 67.4% higher odds of AD than non-drinkers (OR = 1.67, 95% CI: [1.17, 2.41], *p* = 5.34 × 10^–3^). Detailed characteristics of tea intake and AD of 275,285 participants were summarized in Supplementary Table 5. Though, we did not find significant association between tea intake and PRS of AD, the overall tendency of the risk of AD predicted by PRS increased as the amount of tea intake increased (Supplementary Figure 1).

## Discussion

The two-sample Mendelian randomization analysis found that an extra cup of tea consumed per day was significantly associated with the risk of AD. The association analysis has a consistent finding when the number of cups per day of tea exceeds six. In addition, we found that the number of cups of tea consumed per day was inversely associated with gray matter volume and right hippocampus volume but positively associated with whiter matter volume and total brain volume. Other than that, we did not find a causal association between tea intake and WMH volume, MD, FA, BMBs, and left hippocampus volume.

This MR study satisfied the three assumptions of MR analysis. First, we selected genetic instruments using a relative strict cutoff value and the F-statistics of the selected SNPs were greater than 10, which ensures the genetic variants were strongly associated with the exposures. Secondly, after checking the SNPs at the phenoscanner, we rules out the possibility that the genetic variants may be associated with confounders. The MR-Egger test did not find pleiotropy in both primary analysis and secondary analysis, which suggested no violation of pleiotropy. Though, the power of the analysis could be decreased by the small proportion of variance explained, since we have large F-statistics, the power of the analysis was not be greatly influenced.

Our MR analysis revealed a risky effect of tea intake on AD. A nominal risk effect of tea intake was found that one extra cup of tea consumed per day would dramatically increase the risk of AD. Similarly, a recent two-sample MR study showed that per extra cup of coffee was associated with an increased risk of AD (35). A possible explanation of the risk effect could be the caffeine in tea. A study of long-term low-dose caffeine effects on an AD mice model indicates that caffeine was associated with increased anxiety behaviors, which might increase the risk of AD (36). Other observational studies suggest that caffeine can cause sleep loss, prolong sleep latency, and induce insomnia, which contributes to cognitive decline, and might also heighten the risk of AD by increasing the β-amyloid burden (37–41).

In addition, a nonlinear relationship between tea intake and AD was reported by the association analysis, suggesting daily consumption of 5–6 cups to be the most protective and indicating possible increased risk effects for daily consumption of greater than 13 cups of tea. Similarly, a recent cohort study on UK Biobank also finds a nonlinear relationship between tea intake and risk of dementia and suggested daily consumption of three cups of tea to be the strongest protective (42). However, due to the non-significant results in the association between tea intake and PRS of AD, the amount of tea intake to reduce risk of AD still needs further exploitation.

We further detected that the amount of tea consumed was associated with brain volumes, which may give clues on how tea intake affects AD. Tea intake was inversely related to gray matter and right hippocampus volume, and positively associated with total brain and white matter volume. Other studies have found that diet or eating behaviors could impact brain volumes (43–45). For example, a recent Mendelian randomization study found inverse association between coffee consumption and gray matter volume (46). It has been suggested that a larger brain volume provides a greater cerebral reserve against the effects of AD, maintaining cognitive function in the presence of neurodegeneration (47). Thus, our results showed that the risk effects of tea intake on AD were contributed by its decreasing effect on gray matter volume. Gray matter volume reduction is a prominent AD feature, indicating neuron loss and subsequent cognitive decline (48). A double-blinded randomized trial reports that participants with regulatory 10-day caffeine intake significantly reduced gray matter volume, suggesting that the decreasing effect of tea intake on gray matter volume in our study may be contributed by caffeine. Meanwhile, our results also showed that increasing cups of tea consumed per day could lead to an increased risk of right hippocampus loss, which also supported the results that increasing the amount of tea intake could lead to an increased risk of AD. Gray matter volume and right hippocampal volume have different brain functions. Decreasing gray matter volume was associated with lower working memory performance in AD patients (49). Right hippocampal volume loss affected auditory verbal learning test performance in patients with AD (50). Therefore, tea intake may increase the risk of AD by reducing the working memory ability as well as language functions.

There are four main strengths of the present study. First, our study is the multiple dimensional investigations of the associations between tea consumption and AD, including association analysis and causal inference using two-sample MR with two phenotypes of tea consumption. Using large-scale GWAS summary statistics from IGAP and UK Biobank rules out potential instrument biases and ensures the credibility and validity of our results. Therefore, our conclusion that tea consumption was associated with an increased risk of AD could be considered comprehensive and robust based on the converging results from different approaches. Second, the main conclusion of causality of tea intake on AD was significant in IVW and WM methods (*p* < 0.05) and is also weak significant in MR-Egger regression (*p* < 0.1). Also, the primary analysis results passed all the sensitivity analyses, including heterogeneity, pleiotropy, and outliers, demonstrating our results’ robustness and reliability. Meanwhile, when using the conventional genome significance level *p* < 5 × 10^–8^ to select genetic instruments, the results still showed that tea intake was significantly associated with increased odds of AD in three methods (OR_IVW_ = 1.48, 95% CI: [1.08, 2.02]; OR_MR–Egger_ = 2.10, 95% CI: [1.02, 4.31]; OR_WM_ = 2.01, 95% CI: [1.22, 3.31]) and passed all the sensitivity analysis (Supplementary Table 6). A consistent finding from different methods makes the finding less likely to be a false positive in MR analysis. Third, our primary analysis and significant secondary analysis had enough power of the analysis to generate the causal estimates. Finally, we further investigated the potential mechanisms or pathways, such as brain volumes and CSVD, using MRI markers that might explain how tea consumption could impact AD. The application of the Bonferroni correction for multiple testing in the secondary outcomes provides more confidence in terms of the potential involvement of tea consumption in CSVD and brain volumes that are significantly associated with AD.

There are some limitations in our analyses. First, the MR analysis of tea consumption and AD was insignificant using an independent AD dataset as replication. However, the overall direction of the replicated MR analyses matched the increasing risk effect of tea consumption. Second, partial sample overlap issues in the exposure and outcome data may bias the results of two-sample MR. The data source of brain volumes, MD, and FA were from UK Biobank (Supplementary Table 7). For these outcomes, we actually applied one-sample MR analysis. However, a recent study showed that the main two-sample MR methods would not be greatly biased by sample overlap and even one-sample MR study design for using data from large biobanks such as the UK Biobank in our study (51); having said that, interpretation based on these results should be cautious. Third, there are a variety of chemical components, such as catechins, theaflavin, gallic acid, chlorogenic acid, ellagic acid, and kaempferol-3-G in tea, that may be related to AD. This study could not conclude which tea components could lead to the increased risk due to the lack of genetic information on these components. Future studies may be conducted to further explore the biological mechanisms underlying the association between tea consumption and AD.

In conclusion, the MR study robustly demonstrated that the genetically predicted tea intake is a causal risk factor for AD and suggested that the risk effects of tea intake on AD may be contributed by decreasing the volume of gray matter and the right hippocampus, which are associated with the decline in language and memory functions. Our results provide more insights into the biological mechanism of tea effect on AD and deliver suggestive guidance of the amount of tea intake per day of tea as an intervention for AD.

## Data availability statement

The original contributions presented in this study are included in the article/Supplementary material, further inquiries can be directed to the corresponding author.

## Author contributions

YS performed the analysis and wrote the manuscript. RS contributed to the conception, designed the research, and revised the manuscript. All authors approved the final manuscript.

## Footnotes

1
https://gwas.mrcieu.ac.uk/

2
http://phenoscanner.medschl.cam.ac.uk

3
https://www.ebi.ac.uk/gwas/
