# Supplementary material for: Extra cup of tea intake associated with increased risk of Alzheimer’s disease: Genetic insights from Mendelian randomization
Source: Front Nutr. 2023 Jan 25;10:1052281. doi: 10.3389/fnut.2023.1052281 (PMC9905237; doi:10.3389/fnut.2023.1052281)
Supplement: Supplementary file 1 [file Data_Sheet_1.docx]

Supplementary Material

Note 1

*Association analysis using binary AD data*

Data on tea intake were collected as part of the UKB touchscreen questionnaire, where participants were asked 'How many cups of tea do you drink a day? (Including black and green tea) (UKB Data-field ID: 1488).

The outcome, Alzheimer's disease (AD), was primarily assessed using hospital inpatient records containing data on admissions and diagnoses obtained from the Hospital Episode Statistics for England, the Scottish Morbidity Record data for Scotland, and the Patient Episode Database for Wales. Diagnoses were recorded using the International Classification of Diseases-10th revision (ICD-10) coding (UKB Data-field ID: 41202). Individuals with AD were identified using ICD-10 codes F00 and G30. In addition to the hospital inpatient records, individuals with self-reported AD were also classified as cases (UKB Data-field ID: 20002). To better fit the model, we restricted the population to European ancestry only and excluded participants aged less than 65 years old. Total of 275,285 participants were included in the model. A generalized logistic regression model was fitted to examine the association between tea intake and AD with people who drink less than 1 cup/day as a reference group adjusted by age at baseline, sex, education, body mass index (BMI), smoking status, alcohol drinking status, total vegetable intake, total fruit intake, total fish intake, sleep duration, and Townsend deprivation index (TDI) .

*Association analysis using PRS of AD*

We want to further analyze the associations between AD and tea intake and, therefore, decided to use genetic predicted polygenic risk score (PRS) to perform association analysis. Summary statistics of AD were obtained from the first stage of the International Genomics of Alzheimer's Project (IGAP), which was used as a base dataset (1). A total of 3,107,973 SNPs were obtained from base data after excluding duplicated SNPs. Individual level genotype-phenotype data were obtained from UK biobank imputed data, which were used as target samples. PRS was derived by PRSice2 using 10,000 permutation tests, and results were derived from a range of *p*-value thresholds for based SNPs (2). After calculating the PRS of AD, a linear regression model was fitted using the PRS of AD and tea intake with people who drink less than 1 cup/day as a reference group adjusted by age at baseline, sex, education, body mass index (BMI), smoking status, alcohol drinking status, total vegetable intake, total fruit intake, total fish intake, sleep duration, and Townsend deprivation index (TDI) .

Before input target samples, quality controls were performed by removing no-sex samples, excluding duplicated SNPs, excluding secondary-degree relatives using kinship, and only keeping European ancestry using PLINK 1.9. All SNPs within 1000kb and with an r^2^ > 0.1 with the target were clumped to adjust for LD in the target population. 30,072,265 SNPs and 377,932 individuals (N_cases_ 2,049/ N_controls_ 375,883) were obtained from UKB imputed data after clumping.

We used the default mode of PRS, in which the PRS were calculated by the number of observed effective allele for each variant multiplied by the corresponding effect size, divided by the number of alleles included in the PRS from that individual. The taking average algorithm of PRS could prevent biased PRS toward more or less genetic markers included in the calculation for that individual (3). Additive genetic model were used as the regression model. To produce better predicted PRS, we also included the first 10 PCs, age and sex, in the PRS generating linear regression model. Nagelkerke pseudo-R2 and significance for the nonzero regression coefficient for PRS were calculated by PRSice-2 with pruning and thresholding (“P +T”) method. PRS of AD at the smallest *p*-value threshold (p = 5×10^-5^) were used as the final scores for regression.

Supplementary Figure 1. Association between PRS of AD and tea intake


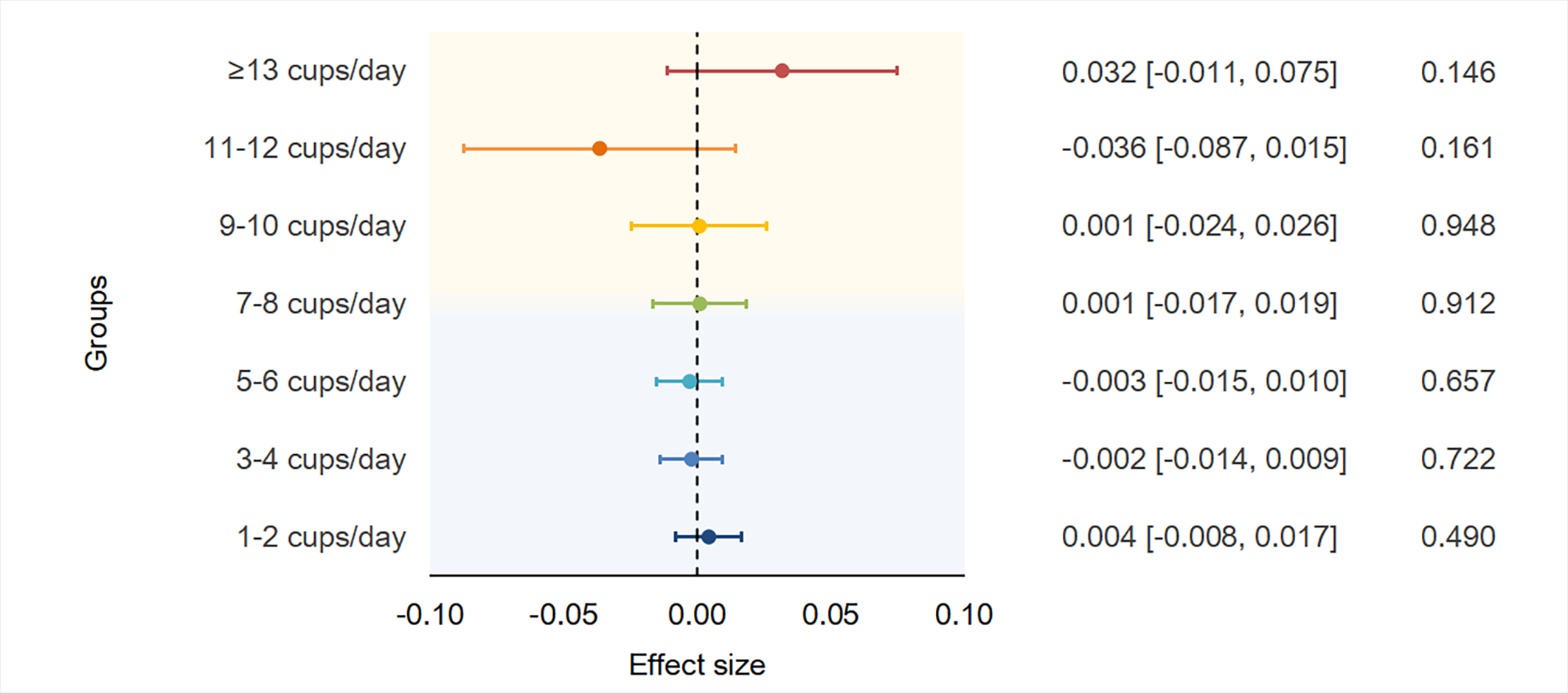


#### Supplementary Table 1. Information of genetic instruments related to tea intake/consumption in the primary analysis.

1. Information of each genetic instruments

| **Exposure** | **SNP** | **Chr** | **Position** | **Effect allele** | **Alter allele** | **Beta** | **SE** | ***p*** | **MAF** | **PVE** | **F-statistics** |
| --- | --- | --- | --- | --- | --- | --- | --- | --- | --- | --- | --- |
| Tea Intake | rs9438548 | 1 | 26757548 | T | C | 0.013 | 0.002 | 1.50E-07 | 0.249 | 6.16E-05 | 27.58 |
|  | rs6593661 | 1 | 95989137 | A | C | -0.016 | 0.003 | 3.10E-07 | 0.137 | 5.86E-05 | 26.21 |
|  | rs111256141 | 1 | 96291273 | A | G | 0.023 | 0.004 | 1.20E-07 | 0.065 | 6.26E-05 | 28.00 |
|  | rs11587444 | 1 | 150722844 | G | A | 0.014 | 0.002 | 1.00E-10 | 0.393 | 9.34E-05 | 41.79 |
|  | rs962242 | 1 | 154592140 | C | T | 0.014 | 0.003 | 1.20E-08 | 0.225 | 7.26E-05 | 32.47 |
|  | rs56188862 | 1 | 174189269 | C | T | -0.016 | 0.002 | 4.30E-13 | 0.387 | 1.17E-04 | 52.50 |
|  | rs12028567 | 1 | 176378828 | C | T | -0.011 | 0.002 | 2.60E-07 | 0.421 | 5.92E-05 | 26.51 |
|  | rs12752825 | 1 | 244015295 | G | A | -0.011 | 0.002 | 4.90E-07 | 0.371 | 5.65E-05 | 25.28 |
|  | rs35970998 | 2 | 40558923 | G | A | -0.013 | 0.002 | 1.40E-07 | 0.263 | 6.21E-05 | 27.78 |
|  | rs10184716 | 2 | 50370382 | T | C | 0.012 | 0.002 | 9.60E-08 | 0.362 | 6.36E-05 | 28.45 |
|  | rs1156588 | 2 | 58515375 | G | A | -0.015 | 0.003 | 2.90E-09 | 0.210 | 7.87E-05 | 35.24 |
|  | rs1868527 | 3 | 42257513 | G | A | 0.013 | 0.002 | 2.70E-07 | 0.248 | 5.84E-05 | 26.46 |
|  | rs57462170 | 3 | 50239803 | A | G | 0.019 | 0.003 | 1.90E-08 | 0.109 | 5.91E-05 | 31.62 |
|  | rs2117137 | 3 | 89525505 | G | A | 0.013 | 0.002 | 1.70E-09 | 0.405 | 7.07E-05 | 36.34 |
|  | rs111285082 | 4 | 2142094 | A | G | -0.024 | 0.005 | 1.90E-07 | 0.055 | 8.12E-05 | 27.18 |
|  | rs1481012 | 4 | 89039082 | G | A | -0.026 | 0.003 | 5.30E-15 | 0.112 | 6.07E-05 | 61.15 |
|  | rs114002124 | 4 | 134454874 | A | G | -0.025 | 0.005 | 5.60E-08 | 0.060 | 1.37E-04 | 29.49 |
|  | rs4862411 | 4 | 185664774 | C | T | 0.013 | 0.003 | 4.00E-07 | 0.234 | 6.59E-05 | 25.69 |
|  | rs2403304 | 5 | 103733250 | C | T | -0.011 | 0.002 | 4.30E-07 | 0.371 | 5.74E-05 | 25.55 |
|  | rs72797284 | 5 | 152031650 | G | A | -0.017 | 0.002 | 7.00E-13 | 0.271 | 5.71E-05 | 51.56 |
|  | rs11752836 | 6 | 11492836 | G | A | 0.011 | 0.002 | 2.10E-07 | 0.464 | 1.15E-04 | 26.98 |
|  | rs2478875 | 6 | 51283110 | G | A | 0.022 | 0.003 | 5.10E-17 | 0.209 | 6.03E-05 | 70.30 |
|  | rs7757102 | 6 | 137222671 | G | A | -0.012 | 0.002 | 3.10E-08 | 0.445 | 6.40E-05 | 30.62 |
|  | rs17655642 | 7 | 13329118 | C | T | 0.013 | 0.002 | 4.10E-07 | 0.237 | 1.57E-04 | 25.63 |
|  | rs4410790 | 7 | 17284577 | C | T | 0.041 | 0.002 | 3.40E-76 | 0.369 | 5.75E-05 | 341.27 |
|  | rs9648476 | 7 | 39293033 | A | G | 0.013 | 0.002 | 1.10E-08 | 0.377 | 7.30E-05 | 32.72 |
|  | rs17685 | 7 | 75616105 | A | G | 0.023 | 0.002 | 1.60E-22 | 0.278 | 6.84E-05 | 95.36 |
|  | rs7012814 | 8 | 9173358 | A | G | -0.011 | 0.002 | 3.80E-07 | 0.474 | 5.73E-05 | 25.77 |
|  | rs2409654 | 8 | 10676935 | T | C | 0.012 | 0.002 | 4.40E-07 | 0.288 | 7.62E-04 | 25.49 |
|  | rs13282783 | 8 | 22088975 | T | C | -0.014 | 0.002 | 7.90E-09 | 0.286 | 7.99E-05 | 33.29 |
|  | rs7825157 | 8 | 93177709 | T | C | -0.013 | 0.003 | 2.40E-07 | 0.234 | 7.31E-05 | 26.66 |
|  | rs72748419 | 9 | 81821528 | G | T | 0.027 | 0.005 | 8.70E-08 | 0.047 | 2.13E-04 | 28.64 |
|  | rs56019762 | 10 | 10205581 | A | G | -0.012 | 0.002 | 2.90E-07 | 0.265 | 8.62E-05 | 26.33 |
|  | rs10752269 | 10 | 12692902 | A | G | -0.013 | 0.002 | 1.30E-09 | 0.494 | 5.76E-05 | 36.88 |
|  | rs2351187 | 10 | 86850616 | A | G | 0.013 | 0.002 | 1.60E-08 | 0.319 | 5.70E-05 | 31.96 |
|  | rs2351822 | 10 | 87448297 | A | C | -0.011 | 0.002 | 2.40E-07 | 0.495 | 7.44E-05 | 26.66 |
|  | rs4418728 | 10 | 94839724 | T | G | -0.012 | 0.002 | 3.70E-08 | 0.451 | 5.96E-05 | 30.31 |
|  | rs10764990 | 10 | 129152608 | A | G | -0.012 | 0.002 | 1.90E-08 | 0.393 | 7.55E-05 | 31.59 |
|  | rs17245213 | 11 | 1679769 | A | G | -0.015 | 0.003 | 2.00E-08 | 0.208 | 6.40E-05 | 31.52 |
|  | rs11022752 | 11 | 13307622 | G | A | 0.013 | 0.002 | 2.50E-08 | 0.269 | 5.88E-05 | 31.10 |
|  | rs10741694 | 11 | 16286183 | C | T | 0.015 | 0.002 | 7.90E-12 | 0.372 | 8.24E-05 | 46.78 |
|  | rs6265 | 11 | 27679916 | T | C | -0.014 | 0.003 | 1.90E-07 | 0.188 | 5.74E-05 | 27.10 |
|  | rs901746 | 11 | 47260319 | G | A | 0.013 | 0.002 | 5.60E-08 | 0.302 | 7.14E-05 | 29.51 |
|  | rs603826 | 11 | 118970878 | C | A | 0.012 | 0.002 | 7.80E-08 | 0.371 | 5.96E-05 | 28.87 |
|  | rs977474 | 12 | 11284772 | T | C | 0.022 | 0.003 | 2.40E-14 | 0.166 | 6.77E-05 | 58.18 |
|  | rs2160515 | 12 | 16753965 | G | A | -0.011 | 0.002 | 2.50E-07 | 0.437 | 7.06E-05 | 26.62 |
|  | rs6488854 | 12 | 17055470 | G | A | 0.011 | 0.002 | 2.70E-07 | 0.378 | 7.04E-05 | 26.48 |
|  | rs10082992 | 12 | 72826693 | A | C | -0.013 | 0.002 | 1.60E-07 | 0.244 | 6.95E-05 | 27.44 |
|  | rs2645929 | 13 | 56444529 | G | A | -0.015 | 0.003 | 3.50E-08 | 0.187 | 1.05E-04 | 30.42 |
|  | rs7999399 | 13 | 89233505 | T | C | 0.012 | 0.002 | 4.00E-08 | 0.444 | 6.06E-05 | 30.13 |
|  | rs17576658 | 13 | 100272019 | A | G | -0.013 | 0.002 | 4.10E-08 | 0.247 | 6.59E-05 | 30.12 |
|  | rs6829 | 13 | 111531264 | T | C | -0.012 | 0.002 | 3.70E-08 | 0.404 | 7.86E-05 | 30.28 |
|  | rs12591786 | 15 | 60902512 | T | C | -0.018 | 0.003 | 3.70E-10 | 0.159 | 6.45E-05 | 39.27 |
|  | rs2472297 | 15 | 75027880 | T | C | 0.053 | 0.002 | 2.30E-109 | 0.262 | 1.30E-04 | 493.64 |
|  | rs224240 | 16 | 3316340 | A | G | -0.013 | 0.002 | 6.90E-08 | 0.241 | 5.95E-05 | 29.08 |
|  | rs9937354 | 16 | 53799847 | A | G | -0.014 | 0.002 | 4.90E-11 | 0.424 | 5.92E-05 | 43.23 |
|  | rs12446615 | 16 | 73915896 | G | A | -0.011 | 0.002 | 6.50E-08 | 0.433 | 6.13E-05 | 29.20 |
|  | rs2279844 | 17 | 40819809 | A | G | -0.012 | 0.002 | 4.00E-08 | 0.379 | 6.80E-05 | 30.15 |
|  | rs1285244 | 17 | 77797634 | G | A | 0.011 | 0.002 | 4.40E-07 | 0.463 | 6.76E-05 | 25.49 |
|  | rs62092408 | 18 | 52753099 | A | G | 0.015 | 0.003 | 8.90E-08 | 0.191 | 6.73E-05 | 28.60 |
|  | rs57631352 | 19 | 4338173 | G | A | -0.013 | 0.002 | 1.70E-08 | 0.297 | 6.73E-05 | 31.87 |
|  | rs4808193 | 19 | 19410622 | C | T | 0.015 | 0.002 | 1.70E-11 | 0.335 | 6.77E-05 | 45.24 |
|  | rs1433071 | 19 | 30913098 | G | T | -0.011 | 0.002 | 3.40E-07 | 0.453 | 6.42E-05 | 26.02 |
|  | rs6033239 | 20 | 11845930 | T | G | -0.012 | 0.002 | 1.80E-07 | 0.362 | 8.78E-05 | 27.23 |
|  | rs455400 | 21 | 31312418 | G | A | -0.014 | 0.003 | 4.60E-07 | 0.165 | 1.10E-03 | 25.44 |
|  | rs4817505 | 21 | 34343828 | C | T | 0.015 | 0.002 | 4.20E-12 | 0.390 | 6.12E-05 | 48.01 |
|  | rs2839188 | 21 | 47692798 | T | C | 0.012 | 0.002 | 5.90E-08 | 0.378 | 6.50E-05 | 29.40 |
|  | rs9624470 | 22 | 24820268 | A | G | 0.025 | 0.002 | 1.30E-31 | 0.420 | 9.66E-05 | 136.84 |
| Tea consumed | rs4905945 | 14 | 100777793 | C | T | 0.012 | 0.002 | 4.70E-07 | 0.363 | 5.93E-05 | 25.39 |
|  | rs7276934 | 21 | 37872250 | A | G | 0.014 | 0.003 | 2.80E-08 | 0.323 | 6.89E-05 | 30.81 |

1. Total PVE for each exposure

| **Exposures** | **No.SNPs** | **Total PVE (%)** |
| --- | --- | --- |
| Tea intake | 68 | 0.68 |
| Tea consumed | 2 | 0.01 |

PVE: proportion of variance explained. PVE was calculated as: R^2^ = (reported GWAS effect)^2^ × 2 × minor allele frequency × (1 – minor allele frequency) / variance of trait. F statistics per variant was calculated as: (Sample size – 2) × R^2^ / (1 – R^2^).

Supplementary Table 2. Power analysis results. AD: Alzheimer’s disease; PVE: proportion of variance explained.

| **Outcome** | **Sample size** | **OR** | **PVE** | **Power** |
| --- | --- | --- | --- | --- |
| **AD** | 54,162 | 1.48 | 0.71% | 0.97 |
| **Brain Microbleeds** |  |  |  |  |
| Any | 3556 | 0.12 | 0.78% | 0.015 |
| Lobar | 2179 | 0.24 | 0.77% | 0.034 |
| Mixed | 1293 | -0.03 | 0.76% | 0.03 |
| **Brain Volume** |  |  |  |  |
| Total Brain Volume | 33224 | 1.89 | 0.03% | 1 |
| Grey Matter Volume | 33224 | -1.73 | 0.03% | 0.99 |
| White Matter Volume | 33224 | 1.9 | 0.02% | 0.99 |
| Left Hippocampus Volume | 33224 | -0.26 | 0.02% | 0.01 |
| Right Hippocampus Volume | 33224 | -0.86 | 0.02% | 0.33 |
| **CSVD** |  |  |  |  |
| White Matter Hyperintensity Volume | 48454 | 0.11 | 0.83% | 0.272 |
| Mean Diffusivity | 17467 | 7.70E-04 | 0.83% | 0.03 |
| Fractional Anisotropy | 17663 | 0.11 | 0.83% | 0.07 |

Note: PVE: proportion of variance explained. PVE was calculated as: R^2^ = (reported GWAS effect)^2^ × 2 × minor allele frequency × (1 – minor allele frequency) / variance of trait.

Supplementary Table 3. Replication analysis of Tea intake and AD

(a) Results of MR analysis using three methods

| **Exposures** | **Method** | **No. Snps** | **OR (95%CI)** | ***p*-value** |
| --- | --- | --- | --- | --- |
| Tea Intake | IVW * | 86 | 1.00 (0.96-1.03) | 0.800 |
|  | MR-Egger | 86 | 1.06 (0.98-1.14) | 0.135 |
|  | WM | 86 | 1.02 (0.97-1.07) | 0.434 |

(b) Sensitivity analysis on replication of tea intake and AD

| **Outcomes** | **MR Egger Intercept Test *p*** | **Heterogeneity *p*** | **MRPRESSO global *p*** |
| --- | --- | --- | --- |
| **AD in replication** | 0.184 | 0.202 | 0.008 |

Supplementary Table 4. MR sensitivity analysis of association between tea intake and primary and secondary outcomes

| **Outcomes** | **MR Egger Intercept Test *p*** | **Heterogeneity *p*** | **MRPRESSO global *p*** |
| --- | --- | --- | --- |
| **AD** | 0.667 | 0.746 | 0.788 |
| **Brain Microbleeds** |  |  |  |
| Any | 0.652 | 0.523 | 0.540 |
| Lobar | 0.322 | 0.929 | 0.931 |
| Mixed | 0.950 | 0.447 | 0.471 |
| **Brain Volume** |  |  |  |
| Total Brain Volume | 0.283 | **8.870**×**10^-5^** | **0.010** |
| Grey Matter Volume | 0.672 | **4.350**×**10^-5^** | **0.010** |
| White Matter Volume | 0.822 | 0.855 | / |
| Left Hippocampus Volume | / | / | / |
| Right Hippocampus Volume | 0.140 | **0.005** | / |
| **CSVD** |  |  |  |
| White Matter Hyperintensity Volume | 0.535 | 0.279 | 0.303 |
| Mean Diffusivity | 0.770 | 0.104 | 0.142 |
| Fractional Anisotropy | 0.260 | **0.020** | **0.019** |
| **Replication AD** | 0.184 | 0.202 | 0.008 |

Supplementary Table 5. Characteristics of Tea intake and AD in UK Biobank Cohort.BMI: Body mass index; TDI: Townsend deprivation index,

| **Characteristic** | **Overall** | **Tea Intake Groups** | | | | | | |  |
| --- | --- | --- | --- | --- | --- | --- | --- | --- | --- |
|  |  | **Less than 1 cup/day** | **1-2 cups/day** | **3-4 cups/day** | **5-6 cups/day** | **7-8 cups/day** | **9-10 cups/day** | **11-12 cups/day** | **≥13 cups /day** |
| **N** | 275,285 | 45,535 | 58,380 | 83,736 | 59,167 | 17,598 | 7,076 | 1,560 | 2,233 |
| **AD (%)** | 1,970 (0.7%) | 353 (0.8%) | 393 (0.7%) | 581 (0.7%) | 414 (0.7%) | 130 (0.7%) | 56 (0.8%) | 10 (0.6%) | 33 (1.5%) |
| **Age (SD)** | 75 (5) | 75 (5) | 75 (5) | 75 (5) | 75 (5) | 75 (5) | 75 (5) | 75 (5) | 75 (5) |
| **Sex (%)** |  |  |  |  |  |  |  |  |  |
| Female | 146,697 (53%) | 25,237 (55%) | 30,042 (51%) | 45,174 (54%) | 31,552 (53%) | 9,600 (55%) | 3,406 (48%) | 692 (44%) | 994 (45%) |
| Male | 128,588 (47%) | 20,298 (45%) | 28,338 (49%) | 38,562 (46%) | 27,615 (47%) | 7,998 (45%) | 3,670 (52%) | 868 (56%) | 1,239 (55%) |
| **Education (%)** |  |  |  |  |  |  |  |  |  |
| College or University degree | 79,121 (29%) | 13,090 (29%) | 19,254 (33%) | 24,496 (30%) | 15,422 (26%) | 4,320 (25%) | 1,695 (24%) | 393 (26%) | 451 (20%) |
| A levels/AS levels or equivalent | 28,137 (10%) | 5,009 (11%) | 6,497 (11%) | 8,379 (10%) | 5,655 (9.7%) | 1,671 (9.6%) | 600 (8.6%) | 142 (9.2%) | 184 (8.3%) |
| O levels/GCSEs or equivalent | 58,294 (21%) | 9,932 (22%) | 12,570 (22%) | 17,940 (22%) | 12,300 (21%) | 3,623 (21%) | 1,297 (19%) | 269 (17%) | 363 (16%) |
| CSEs or equivalent | 9,608 (3.5%) | 1,559 (3.5%) | 1,842 (3.2%) | 2,927 (3.5%) | 2,243 (3.8%) | 633 (3.6%) | 275 (3.9%) | 50 (3.3%) | 79 (3.6%) |
| NVQ or HND or HNC or equivalent | 19,977 (7.3%) | 3,215 (7.1%) | 3,767 (6.5%) | 5,877 (7.1%) | 4,772 (8.2%) | 1,411 (8.1%) | 603 (8.6%) | 146 (9.5%) | 186 (8.4%) |
| Other professional qualifications eg: nursing, teaching | 16,897 (6.2%) | 2,806 (6.2%) | 3,370 (5.8%) | 5,163 (6.2%) | 3,743 (6.4%) | 1,150 (6.6%) | 442 (6.3%) | 80 (5.2%) | 143 (6.5%) |
| None of the above | 60,316 (22%) | 9,472 (21%) | 10,529 (18%) | 18,062 (22%) | 14,333 (25%) | 4,586 (26%) | 2,078 (30%) | 458 (30%) | 798 (36%) |
| **BMI (SD)** | 27.5 (4.7) | 28.2 (5.1) | 27.4 (4.6) | 27.3 (4.5) | 27.5 (4.6) | 27.5 (4.6) | 27.9 (4.8) | 27.8 (4.8) | 27.7 (4.8) |
| **Smoking status (%)** |  |  |  |  |  |  |  |  |  |
| Never | 143,379 (52%) | 22,412 (49%) | 30,390 (52%) | 45,490 (54%) | 31,432 (53%) | 8,871 (50%) | 3,181 (45%) | 657 (42%) | 946 (42%) |
| Previous | 106,849 (39%) | 17,737 (39%) | 23,373 (40%) | 32,502 (39%) | 22,620 (38%) | 6,646 (38%) | 2,617 (37%) | 586 (38%) | 768 (34%) |
| Current | 23,933 (8.7%) | 5,211 (11%) | 4,407 (7.5%) | 5,420 (6.5%) | 4,842 (8.2%) | 1,998 (11%) | 1,240 (18%) | 310 (20%) | 505 (23%) |
| Prefer not to answer | 1,124 (0.4%) | 175 (0.4%) | 210 (0.4%) | 324 (0.4%) | 273 (0.5%) | 83 (0.5%) | 38 (0.5%) | 7 (0.4%) | 14 (0.6%) |
| **Alcohol drinking (%)** |  |  |  |  |  |  |  |  |  |
| Never | 9,471 (3.4%) | 2,052 (4.5%) | 1,428 (2.4%) | 2,445 (2.9%) | 2,148 (3.6%) | 814 (4.6%) | 361 (5.1%) | 92 (5.9%) | 131 (5.9%) |
| Previous | 9,870 (3.6%) | 2,229 (4.9%) | 1,573 (2.7%) | 2,304 (2.8%) | 2,211 (3.7%) | 796 (4.5%) | 438 (6.2%) | 121 (7.8%) | 198 (8.9%) |
| Current | 255,731 (93%) | 41,212 (91%) | 55,338 (95%) | 78,934 (94%) | 54,760 (93%) | 15,971 (91%) | 6,269 (89%) | 1,344 (86%) | 1,903 (85%) |
| Prefer not to answer | 213 (<0.1%) | 42 (<0.1%) | 41 (<0.1%) | 53 (<0.1%) | 48 (<0.1%) | 17 (<0.1%) | 8 (0.1%) | 3 (0.2%) | 1 (<0.1%) |
| **Sleep duration (SD)** | 7 (1) | 7 (1) | 7 (1) | 7 (1) | 7 (1) | 7 (1) | 7 (1) | 7 (2) | 7 (2) |
| **Total vegetable intake** | 4 (5) | 4 (5) | 4 (4) | 4 (4) | 4 (4) | 4 (5) | 4 (5) | 4 (6) | 4 (5) |
| **Total fruit intake** | 2 (5) | 2 (5) | 2 (5) | 2 (5) | 2 (5) | 2 (5) | 2 (5) | 2 (5) | 2 (5) |
| **Total fish intake** | 4 (1) | 3 (1) | 4 (1) | 4 (1) | 4 (1) | 3 (1) | 3 (2) | 3 (2) | 3 (2) |
| **TDI** | -1.67 (2.89) | -1.46 (2.99) | -1.77 (2.84) | -1.83 (2.80) | -1.69 (2.86) | -1.51 (2.97) | -1.09 (3.16) | -1.09 (3.18) | -0.68 (3.35) |

Supplementary Table 6. MR analysis between tea intake and AD using *p* < 5×10^-8^ for SNP selection

(a) Results of MR analysis using three methods

| **Exposure** | **Method** | **No.snps** | **OR [95%CI]** | ***p*-value** |
| --- | --- | --- | --- | --- |
| Tea intake | IVW | 35 | 1.48 [1.08, 2.02] | 1.47×10^-2^ |
|  | MR-Egger | 35 | 2.10 [1.02, 4.31] | 5.27×10^-2^ |
|  | WM | 35 | 2.01 [1.22, 3.31] | 6.24×10^-3^ |

(b) Sensitivity analysis using *p* < 5×10^-8^ for SNP selection

| **Outcome** | **MR Egger Intercept Test *p*** | **Heterogeneity *p*** | **MRPRESSO global *p*** |
| --- | --- | --- | --- |
| **AD** | 0.292 | 0.690 | 0.691 |

Supplementary Table 7 Data sources of MR analysis.

| **Outcomes** | **Data Source** | **Reference** |
| --- | --- | --- |
| AD (Primary analysis) | IGAP first stage | (1) |
| AD (replication) | Meta-analysis including **UK Biobank** | (4) |
| Brain volumes | **UK Biobank** | (5) |
| Brain microbleeds | the Cohorts of Heart and Aging Research in Genomic Epidemiology (CHARGE) consortium and the **UK Biobank**, combined with additional data from the case–control Alzheimer's Disease Neuroimaging Initiative (ADNI) database and the Massachusetts General Hospital Genes Affecting Stroke Risk and Outcomes Study (MGH-GASROS) and Clinical Relevance of Microbleeds in Stroke due to Atrial Fibrillation (CROMIS-2 AF) stroke studies | (6) |
| MD | **UK Biobank** | (7) |
| FA | **UK Biobank** | (7) |
| WMH | meta-analysis combining samples from the Cohorts for Heart and Aging Research in Genomic Epidemiology (CHARGE) Consortium and the **UK Biobank** | (8) |

Note: AD: Alzheimer’s disease; MD: Mean diffusivity; FA: Frictional anisotropy; WMH: White matter hypertensities.

**References:**

1. Kunkle BW, Grenier-Boley B, Sims R, Bis JC, Damotte V, Naj AC, et al. Genetic meta-analysis of diagnosed Alzheimer’s disease identifies new risk loci and implicates Aβ, tau, immunity and lipid processing. Nature genetics. 2019;51(3):414-30.

2. Choi SW, O'Reilly PF. PRSice-2: Polygenic Risk Score software for biobank-scale data. Gigascience. 2019;8(7):giz082.

3. Li J, Chaudhary DP, Khan A, Griessenauer C, Carey DJ, Zand R, et al. Polygenic risk scores augment stroke subtyping. Neurology Genetics. 2021;7(2).

4. Wightman DP, Jansen IE, Savage JE, Shadrin AA, Bahrami S, Holland D, et al. A genome-wide association study with 1,126,563 individuals identifies new risk loci for Alzheimer’s disease. Nature genetics. 2021;53(9):1276-82.

5. Smith SM, Douaud G, Chen W, Hanayik T, Alfaro-Almagro F, Sharp K, et al. An expanded set of genome-wide association studies of brain imaging phenotypes in UK Biobank. Nature neuroscience. 2021;24(5):737-45.

6. Knol MJ, Lu D, Traylor M, Adams HH, Romero JRJ, Smith AV, et al. Association of common genetic variants with brain microbleeds: a genome-wide association study. Neurology. 2020;95(24):e3331-e43.

7. Persyn E, Hanscombe KB, Howson JM, Lewis CM, Traylor M, Markus HS. Genome-wide association study of MRI markers of cerebral small vessel disease in 42,310 participants. Nature communications. 2020;11(1):1-12.

8. Sargurupremraj M, Suzuki H, Jian X, Sarnowski C, Evans TE, Bis JC, et al. Cerebral small vessel disease genomics and its implications across the lifespan. Nature communications. 2020;11(1):1-18.
